# Inhibitory Effect of Curcumol on Jak2-STAT Signal Pathway Molecules of Fibroblast-Like Synoviocytes in Patients with Rheumatoid Arthritis

**DOI:** 10.1155/2012/746426

**Published:** 2012-03-12

**Authors:** Heng Wang, Yongfei Fang, Yong Wang, Zhizhong Wang, Qinghua Zou, Ying Shi, Juerong Chen, Daizhi Peng

**Affiliations:** ^1^Department of Traditional Chinese Medicine, Southwest Hospital, Third Military Medical University, Chongqing 400038, China; ^2^Institute of Burn Research, State Key Laboratory of Trauma, Burns and Combined Injury, Southwest Hospital, Third Military Medical University, Chongqing 400038, China

## Abstract

Hyperplasia of synovial membrane in rheumatoid arthritis (RA) is a critical pathological foundation for inducing articular injury. The janus kinase and signal transducer and activator of transcription (Jak-STAT) pathway plays a critical role in synovial membrane proliferation induced by platelet-derived growth factor (PDGF). To explore the anti-cell proliferation mechanism of curcumol, a pure monomer extracted from Chinese medical plant *zedoary rhizome*, the changes of Jak2-STAT1/3 signal pathway-related molecules in synoviocytes were observed *in vitro*. In this study, the fibroblast-like synoviocytes (FLS) in patients with RA were collected and cultured. The following parameters were measured: cell proliferation (WST-1 assay), cell cycles (fluorescence-activated cell sorting, FACS), STAT1 and STAT3 activities (electrophoretic mobility shift assay, EMSA), and the protein expressions of phosphorylated Jak2, STAT1, and STAT3 (Western blot). It was shown that curcumol could inhibit the RA-FLS proliferation and DNA synthesis induced by PDGF-BB in a dose-dependent manner *in vitro*. The transcription factors activities of STAT1 and STAT3 were obviously elevated after PDGF-BB stimulation (*P* < 0.05). Super-shift experiments identified the STAT1 or STAT3 proteins in the complex. Furthermore, the different concentration curcumol could downregulate the DNA binding activities of STAT1 and STAT3 (*P* < 0.05) and inhibit the phosphorylation of Jak2 while it had no effect on the protein expressions of STAT1 and STAT3. Positive correlations were found between changes of cell proliferation and DNA-binding activities of STAT1 and STAT3, respectively (*P* < 0.01). In conclusion, curcumol might suppress the FLS proliferation and DNA synthesis induced by PDGF-BB through attenuating Jak2 phosphorylation, downregulating STAT1 and STAT3 DNA-binding activities, which could provide theoretical foundation for clinical treatment of RA.

## 1. Introduction

Rheumatoid arthritis (RA) is a chronic destructive disease of the joints that is characterized by chronic proliferative synovitis, infiltration of inflammatory cells into the synovial tissue of joints, and cartilage destruction. Fibroblast-like synoviocytes (FLS) and inflammatory cells, such as macrophages and T cells, produce proinflammatory cytokines, such as interleukin (IL)-1*β* and tumor necrosis factor-*α*, which play key roles in the pathogenesis of RA [1]. In RA, the synovial lining can expand from the normal 1–3 cells to 15 or more cells thickness because of the increased number of synoviocytes. Considering the characteristics of RA synovial tissues such as marked proliferation and invasion to adjacent tissues, comparisons with transformed or neoplastic tissue are natural. RA synovial tissues or cells are not truly malignant, but they have many features of transformation, denoted as “partial transformation”. These features include anchorage-independent growth, loss of contact inhibition, oncogene activation, monoclonal or oligoclonal expansion, detectable telomerase activity, and somatic gene mutations [2, 3]. Therefore, therapies used in the treatment of tumors may be effective in the treatment of RA. Clinical experience shows that RA treatment may be more effective than simple anti-inflammatory therapy if synoviocyte proliferation and pannus production can be suppressed, and apoptosis can be promoted. Pulse therapy of small doses of methotrexate is the gold standard of RA treatment [4]. Therefore, it is of significance to screen effective ingredients from natural antitumor drugs and investigate their effects on synoviocyte proliferation and intrinsic mechanisms involved in RA.

Rhizoma Curcumae (rhizome of *Curcuma*; Ezhu in Chinese) is a traditional Chinese medicine that has been used in removing blood stasis and alleviating pain for over a thousand years in China. The essential oils are considered as active constituents in Rhizoma Curcumae, which include curdione, curcumol, and germacrone. Curcumol is one of the major components of the essential oil of Rhizoma Curcumae with the structure of a guaiane-type sesquiterpenoid hemiketal. It exhibits characteristics such as antitumor, antiproliferation, anti-inflammatory, anti-hepatic fibrosis, antioxidant, and antimicrobial activities with low cytotoxicity [5–8]. The proliferation and RNA synthesis of tumor cell lines derived from female patients including MCF7, MM231, HeLa, and OV-UL-2 were inhibited by curcumol *in vitro* [9]. It was shown that curcumol induced cell death in a dominant apoptotic fashion via the caspases-independent mitochondrial pathway in ASTC-a-1 cells [10]. However, little is known about the characteristics and molecular mechanism of curcumol-induced antiproliferation effect.

In this experiment, we treated synoviocytes of RA with curcumol *in vitro* and evaluated the cytotoxic effects of curcumol on cell viability, cell cycle arrest, signal transducer and activator of transcription (STAT) 1 and STAT3 activities, and protein expressions of phosphorylated Janus kinase (Jak) 2, STAT1, and STAT3. The aim of the study is to explore the effect and mechanism of curcumol on inhibiting cell proliferation at the level of Jak2-STAT signal transduction induced by platelet-derived growth factor (PDGF)-BB.

## 2. Materials and Methods

### 2.1. Materials

Curcumol (C_15_H_24_O_2_) was separated from Curcuma Turmeric Oil (vapor distillated from Rhizoma Curcumae) combining crystal precipitation in lower temperature (0–5°C) with anhydrous ethanol recrystallization and characterized by chromatography with curcumol reference substance (obtained from National Institute for the Control of Pharmaceutical and Biological Products, batch number: CUL081007T, used for assay). The purity of curcumol was greater than 98% by the peak normalization method using HPLC-UV. Working solutions were prepared by dissolving the compound in ethanol before the experiments. Human PDGF-BB was purchased from PeproTech (NJ, USA). WST-1 Cell Proliferation and Cytotoxicity Assay Kit was obtained from KeyGEN (Nanjing, China). Rabbit anti-phospho-Jak2 (Tyr1007/1008), anti-STAT3 polyclonal antibodies were purchased from Biovision (CA, USA). Mouse anti-STAT1 monoclonal antibodies were obtained from BioLegend (CA, USA). LightShift Chemiluminescent EMSA Kit was purchased from Pierce (IL, USA).

### 2.2. Isolation and Culture of Human FLS

The human FLS were isolated from primary synovial tissue obtained from three patients with RA who met the revised American Rheumatism Association criteria [11] and had undergone total joint replacement surgery or synovectomy, as described previously [12]. Briefly, fresh synovial tissues were minced and digested with 0.5 mg/mL of type I collagenase (Sigma-Aldrich, St. Louis, MO, USA) by shaking vigorously at 37°C for 4 h. The supernatant containing the released cells was then removed, and the digestion procedure was repeated two times. Cells were used at passages 4–8, at which time they were comprised of a homogeneous population. Cells were grown at 37°C under a humidified, 5% CO_2_ atmosphere in high glucose-containing Dulbecco's modified eagle's medium (DMEM) (Gibco BRL, Grand Island, NY) supplemented with 10% fetal bovine serum, 2 mmol/L glutamine, 100 units/mL of penicillin, and 100 *μ*g/mL of streptomycin. The study was approved by the Southwest hospital Ethical Committee, and all participants gave written informed consent to their participation.

About 1 × 10^4^ viable cells were added to each well of 96-well cell culture plate for cell proliferation assay, and 1 × 10^6^ cells added to each well of six-well culture plate for the other detections. Cells were randomly divided into several groups: normal control group (normal FLS), PDGF-BB group (25 ng/mL PDGF-BB for 30 min), and the remaining groups were treated with different concentrations of curcumol preinduced by PDGF-BB for 30 min. Curcumol was first dissolved in absolute ethanol, and the resulting solution was diluted with the medium to various concentrations. The final concentration of absolute ethanol should not exceed 1% of the total culture volume. A solvent control group (containing PDGF-BB and 1% absolute ethanol without curcumol) was applied in cell cycle detection. Then, the plate was incubated at 37°C in a 5% CO_2_ atmosphere for 24 h. Cellular nuclear and cytoplasmic proteins were obtained in corresponding methods.

### 2.3. Cell Proliferation Assay

 The FLS were added to each well of 96-well cell culture plate and incubated overnight at 37°C under 5% CO_2_ in a humidified incubator to allow cells to attach to wells. Then, cells were treated with PDGF-BB and different concentrations (25, 50, 100, 200, 400, and 800 *μ*g/mL) of curcumol for 24 h, and the effect of curcumol on the proliferation of cells was determined by WST-1 (4-[3-(4-iodophenyl)-2-(4-nitrophenyl)-2H-5-tetrazolio]-1,3-benzene disulfonate) assay according to the manufacturer's instructions [13, 14].

### 2.4. Cell Cycle Determination

Cell cycle distribution was analyzed using fluorescence-activated cell sorting (FACS). Briefly, after being induced by PDGF-BB and treated with different concentrations (25, 50, 100 *μ*g/mL) of curcumol for 24 h, the cells in six-well plate were harvested, washed twice with PBS, and fixed in 70% ethanol at 4°C for 1 h and centrifuged. The pellet was treated with RNase (20 mg/mL) at room temperature for 30 min and then incubated with propidium iodide (50 mg/mL) for 30 min [10].

### 2.5. Cell Extracts

The FLS in six-well plate were treated with different concentrations (25, 50, 100 *μ*g/mL) of curcumol, and nuclear extracts were prepared by a modification of the methods [15, 16]. Supernatants of cells in culture plate were discarded, and cells were washed three times in cold phosphate-buffered saline (PBS). Nonadherent cells were pelleted and resuspended in 1.5 mL cold PBS. The cells were pelleted for 10 s and resuspended in 100 *μ*L cold Buffer A (10 mmol/L HEPES–NaOH (pH 7.8), 15 mmol/L KCl, 1 mmol/L MgCl_2_, 0.1 mmol/L EDTA, 1 mmol/L dithiothreitol, 1 mmol/L PMSF, and 1 mg/mL leupeptin) by flicking the tube. The cells were allowed to swell on ice for 15 min and then vortexed for 10 s by adding 10% NP-40 10 *μ*L. Samples were centrifuged for 30 s. The pellet was resuspended in 50 *μ*L of cold Buffer B (20 mmol/L HEPES-NaOH (pH 7.9), 1.5 mmol/L MgCl_2_, 0.42 mol/L NaCl, 0.2 mmol/L EDTA, 25% glycerol, 0.5 mmol/L dithiothreitol, 0.5 mmol/L PMSF, and 1 mg/mL leupeptin) and incubated on ice for 30 min for high-salt extraction. Cellular debris was removed by centrifugation for 1 min at 12,000 × *g*, and the supernatant fraction (containing DNA-binding proteins) was stored at −70°C. Protein concentrations were determined by Bradford's method [17].

### 2.6. Electrophoretic Mobility Shift Assay (EMSA) for STAT1 and STAT3 Activities

The assay is based on that DNA-protein complexes migrate slower than unbound DNA or double-stranded oligonucleotides in a native polyacrylamide or agarose gel, resulting in a “shift” in migration of the labeled DNA band. The detection of bands was by “The LightShift Chemiluminescent EMSA kit” that uses a nonisotopic method to detect DNA-protein interactions. Two double-stranded oligonucleotides containing human STAT1 site (P1: 5′-CAT GTT ATG CAT ATT CCT GTA AGT-3′; P2: 5′-ACT TAC AGG AAT ATG CAT AAC ATG -3′) and STAT3 site (P1: 5′-GAT CCT TCT GGG AAT TCC TAG ATC-3′; P2: 5′-GAT CTA GGA ATT CCC AGA AGG ATC-3′) were end-labeled with biotin. Binding assays were performed in 21 *μ*L of binding reaction mixture containing 3 *μ*g nuclear extract protein, DNA-binding buffer and 40 fmol biotin-labeled DNA probe. For super-shift assays, the extracts were incubated first with the STAT1 or STAT3 antibodies for 20 min followed by the addition of the corresponding biotin probe. Reactions were incubated at room temperature for 30 min and analyzed by electrophoresis on a 12% nondenaturing polyacrylamide gel at 200 V for 1.5 h using the high ionic strength conditions. The specificity of binding was confirmed by addition of a 200-fold excess of unlabeled double-stranded oligonucleotides containing human STAT1 or STAT3 site to separate reaction mixtures, respectively. As an additional control, a 200-fold excess of cold oligonucleotide bearing the NF-*κ*B binding site was added to separate reaction mixtures. Competition reactions were incubated for 10 min before addition of labeled oligonucleotide. After transfer the membrane was immediately cross-linked for 15 min on a UV transilluminator equipped with 312 nm bulbs. A chemiluminescent detection method utilizing a luminol/enhancer solution and a stable peroxide solution (Pierce, USA) was used as described by the manufacturer, and membranes were exposed to X-ray films for 2 min before developing. The bands were scanned, and relative intensities were analyzed by Labwork Analysis Software [18, 19].

### 2.7. Western Blot for Protein Expressions of Phosphorylated Jak2 (p-Jak2), STAT1, and STAT3

The FLS in six-well plate were treated with curcumol at the concentrations of 25 *μ*g/mL and 50 *μ*g/mL and lysed in lysis buffer containing 1% Triton X-100, 50 mmol/L HEPES, pH 7.5, 10% glycerol, 150 mmol/L NaCl, 1.5 mmol/L EGTA, 10 mmol/L sodium pyrophosphate, 100 mmol/L NaF, 2 mmol/L sodium orthovanadate, 10 *μ*g/mL aprotinin, and 1 mmol/L phenylmethylsulfonylfluoride. Lysates were cleared by centrifugation at 13,000 × *g* for 15 min at 4°C. After lysis, protein content was quantified by Bradford's method. Equal amounts of protein were loaded on each lane of a SDS-PAGE (sodium dodecyl sulfate polyacrylamide gel electrophoresis) gel and transferred to polyvinylidene fluoride membrane (PVDF) in 25 mmol/L Tris, 192 mmol/L glycine, 20% methanol at 80 mA for 4 h. The filters were blocked to reduce nonspecific binding by incubation in blocking buffer (3% nonfat milk, 0.01 M PBS, 0.02% Tween-20) overnight. The blots were then incubated with rabbit polyclonal antiphosphorylated Jak2 or STAT1 or STAT3 antibody at 1 : 1000 dilution for 1 h, respectively. After being washed with PBS-Tween-20, the blots were incubated with secondary antibody conjugated to horseradish peroxidase blocking buffer for 1 h. The blots were washed extensively with PBS-Tween-20 and developed by the enhanced chemiluminescence (ECL) methods (Pierce, USA) and then exposed to X-ray film for 2 min. The protein signals were quantified by an image-scanning densitometer, and the strength of each protein signal was normalized to the corresponding *β*-actin stain signal [20].

### 2.8. Statistics

 The data were presented as mean ± standard deviations (S.D). Statistical analysis was evaluated by Student's *t*-test (between normal control and PDGF-BB group) or one-way (between PDGF-BB and drug treatments) ANOVA followed by Dunnett's *t*-test for multiple comparisons using SPSS statistical program. A level of *P* < 0.05 was considered statistically significant.

## 3. Results

### 3.1. Effect of Different Concentrations of Curcumol on Cell Proliferation and DNA Synthesis in FLS from Patients with RA

**Figure 1:**
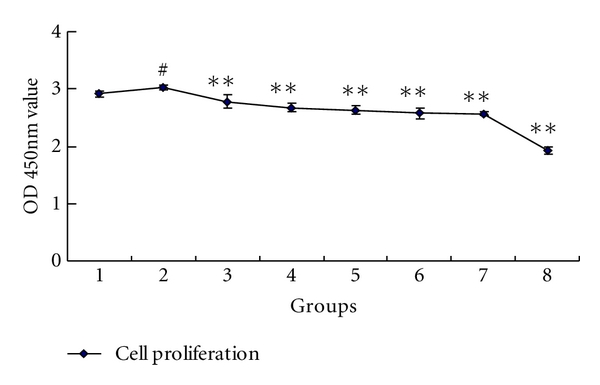
Inhibitory effect of different concentration curcumol on FLS proliferation in patients with RA. 1: normal control; 2: PDGF-BB; 3: curcumol 25 *μ*g/mL; 4: curcumol 50 *μ*g/mL; 5: curcumol 100 *μ*g/mL; 6: curcumol 200 *μ*g/mL; 7: curcumol 400 *μ*g/mL; 8: curcumol 800 *μ*g/mL. Data are expressed as mean ± SD of triplicates. ^#^ *P* < 0.05, ^##^ *P* < 0.01, compared with normal control. *P < 0.05, **P < 0.01, compared with PDGF-BB.

**Figure 2:**
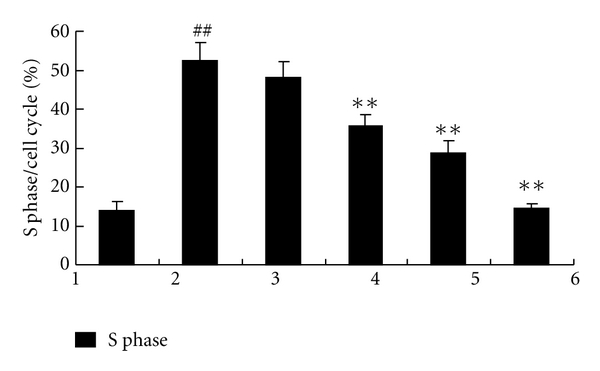
Inhibitory effect of different concentration curcumol on FLS DNA synthesis in patients with RA. 1: normal control; 2: PDGF-BB; 3: solvent control; 4: curcumol 25 *μ*g/mL; 5: curcumol 50 *μ*g/mL; 6: curcumol 100 *μ*g/mL. Data are expressed as mean ± SD of triplicates. ^#^ *P* < 0.05, ^##^ *P* < 0.01, compared with normal control. *P < 0.05, **P < 0.01, compared with PDGF-BB.

Compared to the PDGF-BB group, cell proliferation and DNA synthesis of FLS changed very significantly in the groups treated with curcumol (*P* < 0.01). With the curcumol concentration increasing, the OD value decreased gradually, indicating various degrees of cell proliferation suppression. The inhibitory rate (%) was calculated by the equation: inhibitory  rate  (%) = OD_stimulation_ − OD_curcumol_/OD_stimulation_. The data showed that with the curcumol concentration increasing (25–800 *μ*g/mL), the inhibitory rate of synoviocyte proliferation increased gradually (8.15%, 11.47%, 13.17%, 15.00%, 15.33%, and 36.37%), respectively. We further detected cell cycle using FACS to determine the distribution of cells in G1, G2, and S. It was shown that PDGF-BB stimulation enhanced DNA synthesis compared to the normal control group (52.49% versus 13.87%). The DNA synthesis was not significantly different between the PDGF-BB and solvent control group. With the increasing concentrations of curcumol, the ratio of cells at S phase gradually decreased from 35.76%, 28.74% to 14.52%, demonstrating that curcumol suppressed DNA synthesis of synoviocytes in a concentration-dependent manner. These data showed that various concentrations of curcumol suppressed synoviocyte proliferation and DNA synthesis (Figure 1 and Figure 2). Based on data from preliminary studies, three different concentrations of curcumol (25 *μ*g/mL, 50 *μ*g/mL, and 100 *μ*g/mL) were chosen for the following experiments.

### 3.2. Effect of Different Concentrations of Curcumol on STAT1 and STAT3 Activities in FLS from Patients with RA

**Figure 3:**
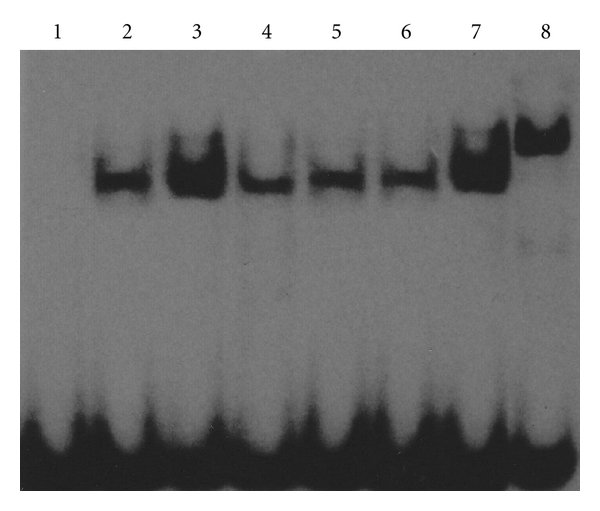
Curcumol inhibited STAT1 DNA-binding activity in FLS from patients with RA. Representative picture of three experiments of EMSA. Lane 1: negative control; lane 2: normal control; lane 3: PDGF-BB; lane 4: curcumol 25 *μ*g/mL; lane 5: curcumol 50 *μ*g/mL; lane 6: curcumol 100 *μ*g/mL; lane 7: IgG control; lane 8: STAT1 antibody.

**Figure 4:**
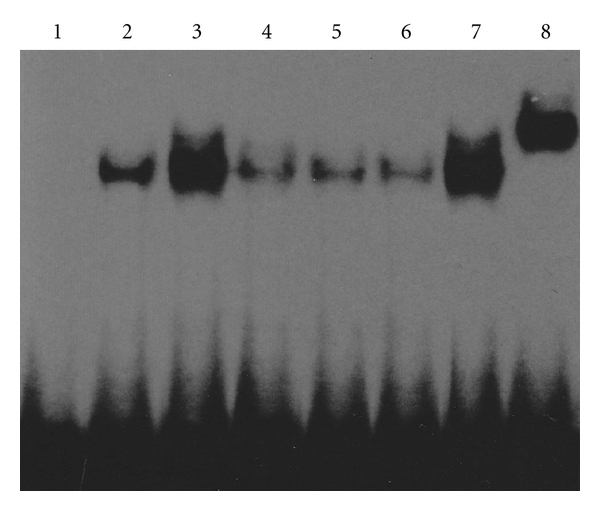
Curcumol inhibited STAT3 DNA-binding activity in FLS from patients with RA. Representative picture of three experiments of EMSA. Lane 1: negative control; lane 2: normal control; lane 3: PDGF-BB; lane 4: curcumol 25 *μ*g/mL; lane 5: curcumol 50 *μ*g/mL; lane 6: curcumol 100 *μ*g/mL; lane 7: IgG control; lane 8: STAT3 antibody.

The specificity of the shift bands in EMSA was verified by competition assays: all the shift bands were suppressed by incubation with a 200-fold excess of unlabeled STAT1 or STAT3 probe and unchanged by competition with a similar amount of another irrelevant oligonucleotide such as NF-*κ*B (data not shown). In absence of stimulation, STAT1 and STAT3 DNA-binding activities of RA synoviocyte were weak in the normal control group. Following stimulation with 25 ng/mL PDGF-BB, STAT1 and STAT3 activities increased significantly (*P* < 0.05). After treatment with various concentrations of curcumol (25 *μ*g/mL, 50 *μ*g/mL, 100 *μ*g/mL), STAT1 activity decreased (*P* < 0.05), and STAT3 activity was suppressed very significantly (*P* < 0.01). In addition, STAT1 and STAT3 antibody super-shift assays showed that the binding bands lagged obviously after addition of STAT1 or STAT3 antibodies, demonstrating the presence of STAT1 or STAT3 proteins in the binding bands (DNA-protein complexes) (Table 1, Figures 3 and 4). Positive correlations were found between changes of cell proliferation and DNA-binding activities of STAT1 and STAT3 following different concentrations of curcumol (*r* = 0.948, *P* = 0.007; *r* = 0.941, *P* = 0.009), respectively.

### 3.3. Effect of Different Concentrations of Curcumol on the Protein Expression of p-Jak2, STAT1, and STAT3 in FLS

**Figure 5:**
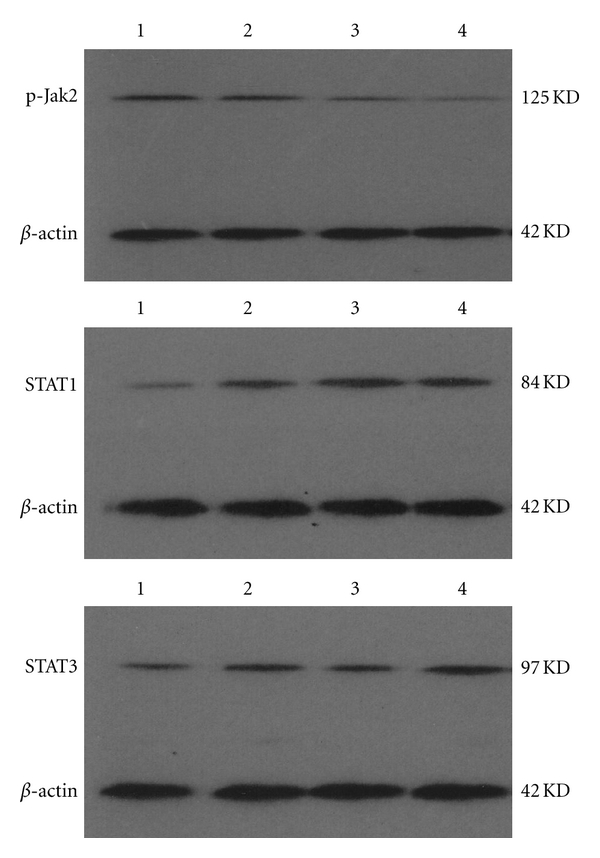
Curcumol inhibited p-Jak2 protein expression of FLS, and the STAT1 and STAT3 protein expression in FLS from patients with RA was not inhibited by curcumol. Representative picture of three experiments of western blot. Lane 1: normal control; lane 2: PDGF-BB; lane 3: curcumol 25 *μ*g/mL; lane 4: curcumol 50 *μ*g/mL.

Western blot analysis showed that p-Jak2 protein was activated in RA synoviocytes and slightly increased following stimulation with 25 ng/mL PDGF-BB (*P* = 0.053). The protein expressions of STAT1 and STAT3 upregulated significantly after PDGF-BB stimulation compared to normal control (*P* = 0.005; *P* = 0.011), respectively. Following treatment with curcumol at the concentration of 50 *μ*g/mL, there were no obvious changes in STAT1 and STAT3 protein expressions (*P* > 0.05) while the phosphorylation of Jak2 was suppressed very significantly (*P* = 0.001) (Table 2 and Figure 5).

## 4. Discussion

Curcumol is an effective anticancer drug used in traditional Chinese medicine. As a potent strategy to protect against carcinogenesis, phytochemicals in Chinese medicine herbs have been applied to protect against carcinogenesis through acting on diverse cellular events including regulation of apoptosis [21]. Curcumol inhibited the proliferation and RNA synthesis of tumor cells (MCF7, MM231, HeLa, and OV-UL-2) *in vitro* while it had no effect on normal mammary gland cells [9]. It has been confirmed that *in vitro* cultured type B synoviocytes from RA patients express oncogenes that are characteristics of actively dividing cells. Hence, the growth of synoviocytes from RA patients is out of control, resulting in excessive proliferation of synovial membrane and pannus invasion [2]. Therefore, RA treatment may refer to method of tumor therapy. Our data demonstrated that certain concentration curcumol ranging from 25 *μ*g/mL to 100 *μ*g/mL could inhibit the RA-FLS proliferation and DNA synthesis induced by PDGF-BB *in vitro*, which showed that curcumol might be one of candidate drugs for treating RA.

The janus kinases (Jaks) have generated great interest in recent years as therapeutic targets due to the unique role that these enzymes play as gatekeepers in the signal transduction process [22–24]. Named after the Roman god of gates and doors (“Janus”), these four enzymes (Jak1, Jak2, Jak3, and Tyk2) control signaling of numerous cytokines and therefore play a central role in acquired and innate immunity and hematopoiesis [25]. The Jak-STAT pathway is the signaling target of a multitude of cytokines, including interferon-*γ*, IL-2, IL-4, IL-6, IL-7, IL-10, IL-12, and IL-15, all of which are thought to have biologically significant roles in rheumatoid synovial inflammation [26, 27]. Seven STATs have been identified, and preliminary work in human synovial tissue suggests that STAT1 expression and activity are increased in RA synovium and STAT3 promotes survival of RA synovial fibroblasts [28–30]. SOCS (suppressors of cytokine signaling) are inhibitory factors in the negative-feedback regulation of Jak-STAT signal pathway [31]. Using retroviral-mediated gene transfer of a dominant negative mutant of Stat3, termed Stat3-YF, it was found that overexpression of Stat3-YF effectively blocked endogenous Stat3 activation and Stat3-dependent gene expression, including expression of the socs3 and myc genes. Stat3-YF-transduced RA synoviocytes failed to grow in culture, exhibited markedly diminished [^3^H] thymidine incorporation, and died spontaneously [30]. These findings demonstrate that STAT1 and STAT3 activation plays an important role in synoviocyte proliferation. In addition, PDGF-BB is one of disulphide-bonded dimeric PDGF isoforms and consists of four polypeptide chains. PDGF receptor (PDGF-R), in turn, may occur as *α* or *β* homodimers or as *α*/*β* heterodimers [32]. The binding of the dimeric ligand to the extracellular portions of the two PDGF-R chains elicits the dimerization of PDGF-Rs and the autophosphorylation of their cytoplasmic tyrosine residues. Receptors for PDGF are abundantly expressed on synoviocytes from patients with RA, and stimulation with PDGF enhances both the anchorage-dependent and -independent growth of synoviocytes. In synovial membrane of RA, synoviocytes, neutrophils, and macrophages produce large amounts of PDGF, leading to cytoplasmic Jak2 tyrosine phosphorylation and STAT1, STAT3 nuclear protein activation through binding to membrane PDGF-R [33, 34]. The present study showed that the transcription factors activities of STAT1 and STAT3 were obviously elevated after PDGF-BB stimulation (*P* < 0.05). Super-shift experiments identified the proteins STAT1 or STAT3 in the complex. Additional treatment with curcumol inhibited the phosphorylation of Jak2 and reduced the DNA-binding activities of STAT1 and STAT3. Furthermore, positive correlations were found between changes of cell proliferation and activities of STAT1 and STAT3 (*P* < 0.01). Accordingly, Jak2-STAT1/3 signal pathway might be the upstream mechanism of the inhibitory effect of curcumol on cell proliferation.

In summary, the potential antiproliferation of curcumol against RA-FLS has been investigated. These results demonstrate for the first time that curcumol might suppress the FLS proliferation and DNA synthesis induced by PDGF-BB through attenuating Jak2 phosphorylation, downregulating STAT1 and STAT3 DNA-binding activities. Although further work is needed to clarify the complicated mechanism of curcumol-induced antiproliferation, curcumol is very promising as a new potential agent for clinical treatment of RA.

At present, different concentrations of curcumol were applied to explore their corresponding mechanisms *in vitro *[6, 9, 10]. There is no literature related to human application *in vivo*, so it is still unknown about curcumol concentrations obtainable in patients. Signal transduction is a biochemical event by which cells transmit a signal from the cell exterior through the cell membrane and into the cytoplasm. Cell proliferation induced by different cytokines involves many related signal molecules, including receptors, kinases, and secondary messengers [35].Therefore, it is necessary to further explore the effects of curcumol on FLS proliferation induced by other cytokines (tumor necrosis factor-alpha, basic fibroblast growth factor, and transforming growth factor-beta) in the future.

## Figures and Tables

**Table 1:** Effect of different concentrations of curcumol on STAT1 and STAT3 activities in FLS from patients with RA.

Groups	STAT DNA-binding activities	
	STAT1	STAT3
Lane 2 (normal control)	168.75 ± 58.54	176.50 ± 25.51
Lane 3 (PDGF-BB 25 ng/mL)	296.75 ± 72.96^#^	344.50 ± 50.98^#^
Lane 4 (curcumol 25 *μ*g/mL)	90.65 ± 14.94*	116.38 ± 17.81**
Lane 5 (curcumol 50 *μ*g/mL)	109.13 ± 11.86*	96.38 ± 23.24**
Lane 6 (curcumol 100 *μ*g/mL)	87.38 ± 13.24*	81.38 ± 15.56**

Each value represents the mean ± S.D. for three independent experiments. *P < 0.05 versus PDGF-BB. **P < 0.01 versus PDGF-BB (Dunnett's *t*-test). ^#^ *P* < 0.05 versus normal control (Student's *t*-test); one-way ANOVA revealed significant effects between PDGF-BB and drug treatment groups (*F* = 27.823, *P* = 0.000 for STAT1, *F* = 66.587, *P* = 0.000 for STAT3).

**Table 2:** Effect of different concentrations of curcumol on p-Jak2, STAT1, and STAT3 in FLS from patients with RA.

Groups	Normalized density (target protein/*β*-actin)		
	p-Jak2	STAT1	STAT3
Lane 1 (normal control)	0.3282 ± 0.0123	0.1395 ± 0.0009	0.2031 ± 0.0361
Lane 2 (PDGF-BB 25 ng/mL)	0.3886 ± 0.0170	0.3144 ± 0.0117^##^	0.3152 ± 0.0240^#^
Lane 3 (curcumol 25 *μ*g/mL)	0.2042 ± 0.0416	0.4055 ± 0.0865	0.2463 ± 0.0856
Lane 4 (curcumol 50 *μ*g/mL)	0.1128 ± 0.0125**	0.3510 ± 0.0163	0.3909 ± 0.0546

Each value represents the mean ± S.D. for three independent experiments. *P < 0.05 versus PDGF-BB. **P < 0.01 versus PDGF-BB (Dunnett's *t*-test). ^#^ *P* < 0.05 versus normal control. ^##^ *P* < 0.05 versus normal control (Student's *t*-test); one-way ANOVA revealed insignificant effects between PDGF-BB and drug treatment groups (*F* = 2.395, *P* = 0.172 for STAT1, *F* = 4.332, *P* = 0.068 for STAT3).
